# What Quality Suffices for Nanopore Metabarcoding? Reconsidering Methodology and Ectomycorrhizae in Decaying *Fagus sylvatica* Bark as Case Study

**DOI:** 10.3390/jof10100708

**Published:** 2024-10-10

**Authors:** Glen Dierickx, Lowie Tondeleir, Pieter Asselman, Kris Vandekerkhove, Annemieke Verbeken

**Affiliations:** 1 Research Group Mycology, Ghent University, 9000 Gent, Belgium; 2 Research Institute for Nature and Forest, 9500 Geraardsbergen, Belgium

**Keywords:** Nanopore, deadwood, ectomycorrhiza, *Fagus sylvatica*, quality filtering, Phred score, eNano pipeline, natural regeneration, metabarcoding

## Abstract

Nanopore raw read accuracy has improved to over 99%, making it a potential tool for metabarcoding. For broad adoption, guidelines on quality filtering are needed to ensure reliable taxonomic unit recovery. This study aims to provide those guidelines for a fungal metabarcoding context and to apply them to a case study of ectomycorrhizae in the decaying bark of *Fagus sylvatica*. We introduce the eNano pipeline to test two standard metabarcoding approaches: (1) Reference-based mapping leveraging UNITE’s species hypothesis system (SH approach); (2) Constructing 98% OTUs (OTU approach). Our results demonstrate that both approaches are effective with Nanopore data. When using a reference database, we recommend strict mapping criteria rather than Phred-based filtering. Leveraging the SH-system further enhances reproducibility and facilitates cross-study communication. For the 98% OTUs, filtering reads at ≥Q25 is recommended. Our case study reveals that the decay gradient is a primary determinant of community composition and that specific mycorrhizal fungi colonize decaying bark. Complementing our metabarcoding results with root tip morphotypification, we identify *Laccaria amethystina* and *Tomentella sublilacina* as key ectomycorrhizae of saplings on decaying logs. These findings demonstrate that Nanopore sequencing can provide valuable ecological insights and support its broader use in fungal metabarcoding as read quality continues to improve.

## 1. Introduction

### 1.1. Fungal Metabarcoding

A significant hurdle in fungal ecology is that most species elude visual detection. This especially affects community ecological studies where the aim is to include all community members. While second-generation sequencing propelled fungal ecology forward by enabling large-scale metabarcoding, the taxonomic resolution using the dominant Illumina platform is constrained by short amplicon lengths at a maximum of 2 × 300 base-pairs (bp). This hampers the exploitation of the fungal barcode region, the Internal Transcribed Spacer (ITS), which ranges between 250 and 1500 bp [1,2,3]. Third-generation sequencing technologies, such as Nanopore (Oxford Nanopore Technologies Inc., Oxford, UK) and PacBio SMRT (Pacific BioSciences Inc., Menlo Park, CA, USA), have overcome read length limitations, enabling the targeting of the full-length ITS region. While Nanopore initially lagged in quality, advancements in chemistry (V14) and algorithms (transformer model-based architecture, dorado ≥ v0.7) are closing this gap. Raw read accuracy now exceeds 99% correct base call probability [4]. This accuracy surpasses the interspecific distances typically observed for the ITS region [5], making Nanopore a potentially viable tool in fungal metabarcoding.

Despite these advancements, studies on the application of Nanopore sequencing in fungal metabarcoding remain limited, with conclusions varying from unsuitable to feasible for simple communities, depending on the version of the technology used [6,7,8,9,10,11]. A critical step in sequence data processing is quality control. Typically, this involves setting a minimum read quality threshold, expressed in terms of Phred score (Q), which indicates the probability of an incorrect base call. For example, Q10 represents a 90% accuracy (1 in 10 chance of error), while Q30 represents a 99.9% accuracy (1 in 1000 chance of error).

The most widely adopted approach in metabarcoding is generating operational taxonomic units (OTUs) de novo from raw data. However, this method has been considered unfeasible with Nanopore data due to high error rates that significantly impact clustering. Typically, the OTU approach involves setting a minimum Phred score of Q28, as recommended in FASTQC [12], and clustering the filtered reads at a predefined similarity threshold. Currently, enforcing a Q28 threshold results in a near-total reduction of usable data, which has limited the adoption of this approach.

To address the issue of read quality, two approaches have thus far been used. The first approach improves sequence quality by drafting consensus sequences from clusters [6,9,11], as implemented in tools like Decona, ONTrack2, and CONCOMPRA [13,14,15]. While these tools are far more sophisticated than simple clustering, they need fine-tuning of more parameters, adding complexity to their use. Similar to OTU clustering, consensus sequence generation bypasses the need for a reference database, offering an advantage for taxonomic units absent from databases.

The second approach aligns raw reads against a reference database, retaining reads that can be confidently mapped [7,9,10]. This approach depends on the comprehensiveness and accuracy of the reference database and shifts the quality issue to defining what constitutes a confidently mapped read.

In all these approaches, setting a minimum Phred score is standard practice. Therefore, establishing a robust minimal quality threshold for each approach is crucial for the reliable recovery of taxonomic units, which underpins all subsequent analyses. However, guidelines for determining an appropriate threshold are sparse, complicating the analysis of Nanopore data and raising the question of what read quality suffices in the context of metabarcoding.

In this paper, we aim to provide guidelines for the quality filtering of Nanopore metabarcoding data using simple OTU-clustering and reference-based approaches. We detail our experiences with analyzing Nanopore data from a mock community and share our bioinformatics pipeline, eNano.

Building on these insights, we apply our methods to a case study. Specifically, we focus on an often overlooked microsite—the fungal community within decaying bark of European beech (*Fagus sylvatica* L. (1753))—and validate our results through the morphotypification of ectomycorrhizal mantles.

### 1.2. Case Study: Deadwood Bark Ectomycorrhizae

**Figure 1:**
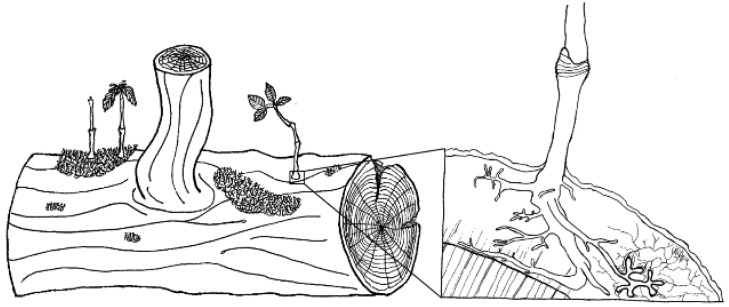
Schematic representation of *Fagus sylvatica* deadwood microsite for regeneration. Seedlings root in the soft, decaying outer layer, the inner bark. Ectomycorrhizal fungi may also proliferate in this substrate, helping seedlings grow.

A specific aspect of habitat creation by deadwood is the case where large logs in intermediate to advanced decay stages act as a tree regeneration site (Figure 1). In mycorrhizal research, these logs are of particular interest because the excavation of root systems can confirm the establishment of ectomycorrhizal fungi (EcM) through morphotypification.

While EcM are abundant in soil and readily mycorrhize roots after germination [16], they are initially absent or scarce in deadwood. Their prevalence increases as the wood decays and becomes more penetrable [17,18,19,20,21]. Eventually, the fungal community in deadwood transitions to resemble that in soil, completing a full ecological transformation [20]. In contrast to fruitbody-based inventories, metabarcoding techniques have detected EcM surprisingly early in the decay process. For example, Rajala et al. [19] found metabolically active EcM in slightly decayed *Picea abies* (L.) H. Karst. (1881) logs, which suggests a continuum of tree regeneration in old natural forests. Several reasons for colonization have been put forward, including nutrient mining [22,23,24], the elevation of fruiting positions, and associating with sapling roots in decaying logs [21,25].

From a seedling’s perspective, associating with EcM is believed to be crucial for survival, especially in environments where nutrients are hard to access, such as recent deadwood [26]. For example, in North America, *Tsuga heterophylla* (Raf.) Sarg. (1899) regenerating on deadwood is known to associate with a wide range of EcM species [16,27,28]. In Europe, Tedersoo et al. [25,29] studied sapling EcM in mixed Estonian forests and found the most frequent species on roots in deadwood to also be common in soil, which is consistent with metabarcoding results.

Over the past decade, we have occasionally observed seedlings of European beech growing on relatively fresh deadwood. These seedlings primarily root in the decaying inner bark—comprising the secondary phloem and nutrient-rich cambium—rather than in the denser wood underneath (Figure 1). This layer, often overlooked except in studies on bark beetles (e.g., [30]), offers a potentially favorable environment for EcM colonization due to its higher moisture content, higher nutrient availability, less compact nature, and faster decay rate [31,32,33,34]. Such conditions could facilitate the penetration of sapling roots and EcM originating either from soil or from propagules. However, there is limited literature available to contextualize our observations, making it an intriguing case study for this underexplored EcM niche.

## 2. Materials and Methods

### 2.1. Study Site and Log Selection

Sampling was conducted in the Joseph Zwaenepoel forest reserve (Brussels, Belgium). This forest reserve is dominated by European beech (*Fagus sylvatica*) and has been left unmanaged since 1983. Further information on the study site can be found in Vandekerkhove et al. [35] and De Keersmaeker et al. [36]. For this study, we specifically searched for large beech logs of intermediate decay stage (Log decay stage 2–3 according to Renvall et al. [37]) bearing newly established saplings. This means that the wood is still mostly hard, and the bark is still present but starts to loosen. We selected 24 suitable logs; an overview of the sampled logs is given in Table S1.

### 2.2. Root Tip Morphotypification and Sanger Barcoding

From the selected logs, a set of 23 beech saplings were collected in September–November 2022 (Table S1). Sapling age was approximated by counting the number of leaf bud scar zones along the dominant axis. To extract saplings from the logs, a knife was used to pry away the outer bark (periderm). Then, the roots were carefully pulled out of the decaying bark (secondary phloem). Saplings were transported separately, with their roots wrapped in aluminum foil to prevent contamination and desiccation of EcM mantles. In addition, five saplings growing on soil in close proximity to the log were collected to serve as a baseline. All plants were stored at 4 °C and processed within three days from collection.

We morphotypified the EcM mantles using the work of Agerer [38,39,40,41,42,43,44]. Mantle coverage was estimated by sectioning the fine root tips (⌀ < 1 mm) into fragments of approximately 5 mm. Each fragment was inspected for mantle presence to estimate total coverage, expressed as the ratio of colonized to total root tips. Mantle micromorphology was assessed using brightfield microscopy. For each morphotype, three root tips were separately preserved for Sanger sequencing.

Kruskal–Wallis rank sum and Dunn’s tests (*stats 4.2.3*, *FSA 0.9.4*) were used to test for differences in EcM species richness and colonization rates between bark- and soil-growing saplings.

Genomic DNA was isolated from the mycorrhized root tips of the collected saplings following a modified cetyltrimethylammonium bromide (CTAB) method [45] Homogenized mantles were suspended in CTAB extraction buffer and incubated on a heat block at 55 °C for at least two hours. The lysate was purified with two rounds of chloroform:isoamyl alcohol cleanups and precipitated using isopropanol (0.54×)|ammonium acetate (0.08×). The ITS region was amplified using Dream Taq Mastermix (Thermo Fisher, Waltham, MA, USA) and primer pair ITS1F/ITS4 (10 µM) [3,46,47]. Amplification conditions were the following: initial denaturation at 94 °C for 5 min; 30 cycles of 94 °C for 30 s, 55 °C for 30 s, and 72 °C for 45 s; and a final extension at 70 °C for 7 min. Amplicons were purified using EXOFastAP (Thermo Fisher, Waltham, MA, USA) and sequenced by Macrogen™ (Amsterdam, The Netherlands).

### 2.3. Nanopore Metabarcoding

#### 2.3.1. Mock Community

We constructed a mock community comprising 16 taxa that represent major ectomycorrhizal lineages and are phylogenetically diverse. Moreover, we included some closely related species to assess the sensitivity of our run at lower taxonomic levels. DNA extracts were sourced from previous collections [48,49,50,51] and opportunistically pooled without prior quality control. Included taxa are detailed in Table S2.

#### 2.3.2. Bark Sampling

We gathered decaying inner bark of 13 beech logs with beech regeneration, with two of these logs sampled at three distinct locations on the log to assess community turnover at the log level. Three logs without regeneration were selected as a control group, resulting in 20 samples for metabarcoding.

We assessed inner bark decay stage (bark DS) on a 3-level scale from early to late, as described in Table 1.

Each substrate sample constitutes a pool of 4 subsamples. Each subsample was taken at a 30° angle from the top of the log, half a meter apart, repeated in two places and on both sides of the log. A sterile knife was used to cut out a 25 cm × 25 cm square in the outer bark layer (periderm) that was carefully peeled back. In each subsample area, three tablespoons of degraded inner bark were added to a sterile Ziplock bag and pooled with other subsamples. The spoon and knife were disinfected between samples by wiping and burning with EtOH. To assess background soil diversity, three soil samples were collected according to the protocol of Tedersoo et al. [52]. All samples were transferred to the lab in a cooling box. Samples were airdried at 40 °C and homogenized by vigorously rubbing in a Ziplock bag. Two grams of sample material were used for DNA extraction.

#### 2.3.3. Wet Lab

Samples were ground under liquid nitrogen. gDNA was isolated as previously described and cleaned using 0.8× SPRI beads (Beckman Coulter, Pasadena, CA, USA).

15 µL PCR reactions were run in duplicate. Each reaction mix consisted of 200 µM dNTPs, 0.5 µM of each primer, 1× Q5 reaction buffer, 0.02 U/µL Q5 HIFI polymerase (New England Biolabs, Ipswich, MA, USA), and 1 µL sample DNA. Cycling conditions were as follows: (1) Initial denaturation for 30 s at 98 °C; (2) 30 cycles of denaturation for 30 s at 98 °C, annealing for 20 s at 60 °C, with an extension for 20 s at 72 °C; (3) Final extension for 2 min at 72 °C. Amplicons were purified using SPRI beads and quantified on a Qubit Fluorometer (Invitrogen, Carlsbad, CA, USA).

Amplicons were normalized to 200 fmol, assuming an average amplicon size of 700 bp, and barcoded using the Native Barcoding kit 96 V14 (SQK-NBD114.96, Oxford Nanopore Technologies Inc., Oxford, UK). 25 fmol was loaded on an R10.4.1 MinION flowcell (Oxford Nanopore Technologies Inc., Oxford, UK). Sequencing ran for 72 h at 260 bp/s.

### 2.4. Bioinformatics

#### 2.4.1. Reference Database

We used the SINTAX-formatted version of UNITEv10 [53]. Fasta header taxonomy annotation was manipulated to include UNITE species hypotheses (SH) by replacing or adding species-level annotations with the RefSeq’s associated SH code (e.g., >UDB05341741;tax=d:Fungi,p:Rozellomycota,s:SH0910702.10FU;). This file can be found on the eNano GitHub page (see Section 2.4.2).

Nine reference sequences were added as dummy SH codes (e.g., SH0000001.10FU) for easier downstream manipulation (Table S2). Three previously generated sanger sequences that did not match any SH. Two references for taxa in the *Russula nigrifacta* species complex: *Russula ustulata* De Lange & Verbeken (2021) as it is part of our mock community, and *R. ambusta* De Lange, Adamčík & F. Hampe (2021) to serve as a control for false positives (GenBank accession number MW172301). In addition, because some of our root tip Sanger sequences did not confidently match any SH, references for *Tomentella sublilacina* (Ellis & Holw.) Wakef. (1962) and *Laccaria amethystina* Cooke (1884) were added. Finally, two large *Laccaria* zOTUs identified in the SH approach were also added (see Section 3.2.4).

#### 2.4.2. eNano Pipeline

Demultiplexed fastq files, as outputted by MinKow (v. 22.10.7), were run through our eNano pipeline, which is opensource and available on the GitHub page of the Research Group Mycology (https://github.com/MycoMatics/eNano (accessed on 4 January 2024)). The pipeline takes a directory with subfolders (each representing sample barcodes) containing fastq files and a SINTAX formatted database as input. It outputs a (LULU-curated) OTU table with assigned taxonomy. Processing steps are detailed in the README file on GitHub. In short, Porechop v. 0.2.4 [54] trims adapters and barcodes, Cutadapt v. ≥ 2.8 [55] reorients sequences and cuts primers, Chopper v. ≥ 0.9 [56] quality filters on average Phred score, Vsearch v. ≥ 2.21 [57] appends sample information and performs clustering, chimera detection, creates a LULU match list and assigns taxonomy with the SINTAX algorithm [58]. LULU-curation [59] is run using the MUMU algorithm v. ≥ 1.0.2 [60], and taxonomic units are aggregated at the species level.

We explored two metabarcoding approaches for processing read data, here explained as the SH approach (reference-based) and the OTU approach (de novo clustering).

#### 2.4.3. SH Approach

Reads were deduplicated into zero-radius Operational Taxonomic Units (zOTUs) and classified into species hypotheses (SH). Given that our mock community comprises databased taxa, which are theoretically classifiable, we established the threshold for the SINTAX classification algorithm by plotting the distribution of SINTAX confidence scores across Phred scores in Figure S1. Using high-quality reads (≥Q28), the SINTAX threshold was determined based on the density of the right tail, which shows an increase starting at 0.80 and a slight dip between 0.90 and 0.95. We therefore set the threshold for singletons at 0.95 and relaxed the criterion for multiton zOTUs to 0.8, considering that such reads are less likely to contain sequencing errors. zOTUs meeting this criterium were retained, and their conspecific abundances were aggregated for each SH. This method prioritizes read classifiability over read quality and is hereafter referred to as the ‘SH approach’.

#### 2.4.4. OTU Approach

Given that not all taxa are represented in databases, and thus, not all reads are classifiable, we also evaluated whether our datasets could support a traditional approach of quality filtering and clustering into 98% OTUs. This method allows the incorporation of sequence clusters that have no obvious match in databases but might be large and influential in driving ecological patterns and is hereafter referred to as the ‘OTU approach’.

#### 2.4.5. Minimum Quality Evaluation

To determine the minimum acceptable quality threshold, we divided the reads from our mock and bark communities based on their average Phred scores. We subsampled Phred-specific datasets to read count at Q28 (4245 mock and 42,560 bark reads per Phred score for Q14–28) and ran eNano on each set. The minimum Phred score for quality filtering was determined as the point where the recovery of taxonomic units (either 98% OTUs or SHs) stabilized. Due to the probabilistic nature of several steps, including chimera removal, clustering, and classification, we ran each dataset one hundred times. Additionally, because singleton exclusion is a common practice in quality filtering but problematic in Nanopore data due to high error rates resulting in many singletons, we also evaluated the effect of singleton taxonomic unit exclusion for both approaches. Finally, to assess species turnover between Phred-specific datasets, Beta-diversity (Bray–Curtis distance) is visualized in Figure S2.

#### 2.4.6. Bark Metabarcoding

Sequence data were processed using insights from both the SH- and OTU approaches. For the SH approach, eNano was run with clustering at 100% identity (--id 1), without a quality threshold (--q 0), using our modified version of the UNITEv10 database. zOTUs were filtered based on SINTAX threshold (0.95 for singletons, 0.8 multitons), and conspecific abundances were aggregated into an SH-table used in all subsequent analyses.

For the OTU approach, eNano was run with a clustering identity of 98% (--id 0.98), a quality threshold of Q25 (--q 25), and a SINTAX threshold of 0.80 (--sintax 0.8) with LULU-curation (--skip-lulu 0). Singleton SHs and OTUs were excluded from analysis.

Tables with taxonomic units were handled using phyloseq 1.42.0 [61], metagMisc 0.5.0 [62], and vegan v. 2.6.4 [63]. Alpha diversity indices—species richness, Shannon-Wiener, and Simpson—were analyzed using a generalized linear model (GLM) with sequencing depth as a covariate to assess variations across bark decay stages. Read abundances were converted to Aitchison distances for Non-metric Multidimensional Scaling (NMDS) and Principal Coordinates Analysis (PCoA). Variable selection for PERMANOVA was conducted through forward and backward selection using AICcPermanova 0.0.2 [64] while considering factors such as decay stages of the log and bark (DS Log, DS Bark) and the presence of regeneration. Indicator species analysis was carried out with the indicspecies package 1.7.13 [65], applying Pearson’s phi coefficient for association (fidelity) and the standard Invdal.g function. Ecological guild assignments were made using FUNGuildR version 0.2.0.9000 [66]. Data processing was performed on the HPC of Ghent University, using eNano v 0.1and R 4.2.3 [67]

## 3. Results

### 3.1. Morphotyping

Saplings growing on logs were primarily found in the intermediate (n = 6) and late (n = 17) stages of bark decay. Although seedlings were occasionally observed in early bark decay stages, their delicate root systems were not successfully retrieved.

For this study, 23 beech saplings growing on 22 distinct logs were selected for root tip morphotyping analysis (Tables S1 and S3). Additionally, five saplings growing in soil were included as a reference group. We constructed ten morphotypes, eight of which were identified by sanger sequencing (Table S4). Two others failed sequencing and were identified based on morphology, the jet-black *Cenococcum geophilum* Fr. (1829) and *Elaphomyces* cf. *muricatus* Fr. (1829), which is recognizable by the spindle shape of the emanating hyphae.

Among the log-based saplings, 13 were colonized by EcM (Table S3). Specifically, two EcM species, *Laccaria amethystina* and *Tomentella sublilacina* were identified, with the former being dominant. In contrast, all soil-grown saplings were colonized by EcM. Also, species diversity is higher in soil, with nine species identified in total (*Laccaria amethystina*, *Lactarius subdulcis* (Pers.) Gray (1821), *Xerocomellus pruinatus* (Fr.) Šutara (2008), *Cenococcum geophilum*, *Russula ochroleuca* Fr. (1829), *Melanogaster* cf. *intermedius* (Berk) Zeller & C.W. Dodge (1937), *Scleroderma citrinum* Pers. (1801), *Inocybe napipes* J.E. Lange (1917) and *Elaphomyces* cf. *muricatus*). EcM coverage (proportion of colonized root tips) and EcM species richness are significantly higher for those saplings growing in soil (Dunn test, resp. *p* = 0.02 and *p* = 0.001). Ten log-based saplings were not colonized; eight of these were estimated to be only two years old. The two older saplings did not show accumulation of EcM, with no EcM present for the 8-year-old sampling and only *Laccaria amethystina* present for the 9-year-old sapling (Table S3).

### 3.2. Metabarcoding

SH and OTU tables for mock and bark datasets can be found in File S1. The negative control was sequenced to account for potential contaminants. Two OTUs mapped to *Malassezia* spp.; these were removed in further analysis.

The average Phred score was 15.3, but it is best re-evaluated after trimming the low-quality, non-informative adapter region. With the first 20 bp trimmed, the average Phred score increased to Q17.3. The median amplicon size was 709 bp.

#### 3.2.1. Minimum Quality Evaluation

**Figure 2:**
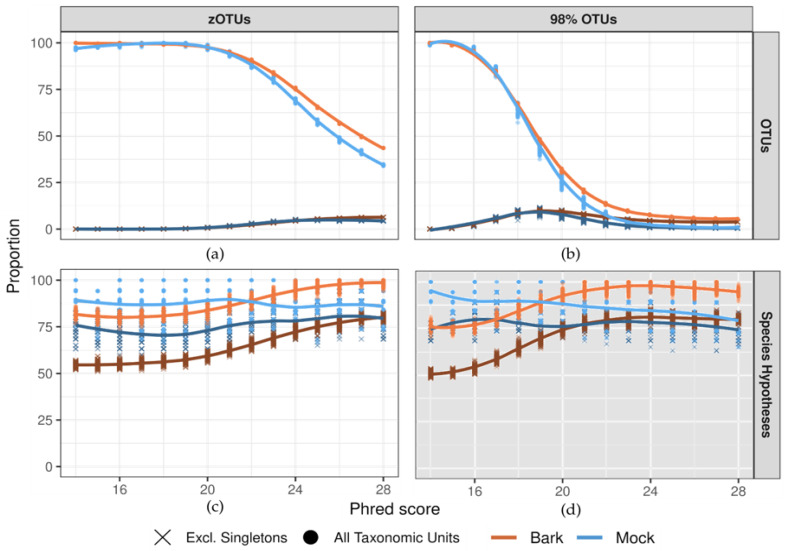
Taxonomic unit recovery using all reads and excluding singleton units (zOTUs, 98% OTUs or SHs), Phred-specific datasets subsampled to amount of reads at Q28. Each datapoint represents 4245 reads for the mock dataset and 42,560 for the bark dataset. Dark and light brown represent Bark data, dark and light blue represent Mock data. Lines represent a loess smoother to visualize trends. (**a**) Intermediate zOTU step in SH approach. (**b**) OTU approach. (**c**) SH approach. (**d**) merging 98% OTUs into SHs was excluded a priori (gray-shaded), shown for completeness. Graphs standardized by maximum recovered units for each analysis and dataset. Nmax-zOTU-bark = 39,382; Nmax-zOTU-mock = 3818; Nmax-98%OTU-bark = 39,348; Nmax-98%OTU-mock = 3722; Nmax-SH-bark = 546; Nmax-SH-mock = 20.

Both approaches demonstrated stabilization in taxonomic unit recovery, with consistent patterns observed for mock and bark datasets. Recovery in the bark community was consistently slower with increasing quality, highlighting the complexity of the dataset (Figure 2b,c).

For the 98% OTU approach, the stabilization in taxonomic unit recovery began at Q24 but never fully plateaued, with 26 and 2605 OTUs recovered at Q28 in the mock and bark communities, respectively (Figure 2b). This trend persisted when excluding singleton OTUs, yielding 15 and 1770 OTUs at Q28.

In the SH approach, the intermediate zOTUs step did not show any signs of stabilizing before Q28, indicating that the sequence error is too impactful to reach a stable number of taxonomic (Figure 2a). When singleton zOTUs were excluded, zOTU recovery slowed at Q25 but never stabilized. At Q28, singleton zOTUs accounted for 28.6% and 37.2% of reads in mock and bark, respectively. The SH approach exhibited stable recovery of SHs across all Phred scores for both datasets, with a slight increase when using higher quality reads in the bark dataset (Figure 2c). At Q28, excluding singleton SHs resulted in fewer SHs in the bark community, reflecting the presence of rare SHs. In the mock community, the average difference when excluding singleton SHs was one SH, *Curvibasidium cygneicollum* (14 vs. 15, Figure 2c).

Merging 98% OTUs into SHs was excluded a priori because clustering at 98% blurs taxonomic resolution well beyond the minimum difference of 0.5% between SHs and the 1.5% clustering threshold of the dynamic UNITE release (Figure 2d).

#### 3.2.2. Mock Community

The OTU approach resulted in 188 OTUs, of which 107 were identified to SH, corresponding to 18 unique SH codes. Fourteen taxa from our input mock were recovered, as well as four contaminant OTUs.

The SH approach generated 127,075 zOTUs, of which 46% could be mapped to SH. 44 SHs were recovered, 17 of which are singletons. 75% of total reads were classified into SHs, and unmatched reads (not detected as fungal) occurred only at Q5–7 (0.27%). Classification rates show a rapid increase up until Q16, after which the increase slows down and finally stabilizes at Q27 (88 ± 0.2% of reads/Phred classified). This pattern was partly repeated for the more complex bark community, whose classification rates stabilized at Q16, as seen in Figure S3.

**Figure 3:**
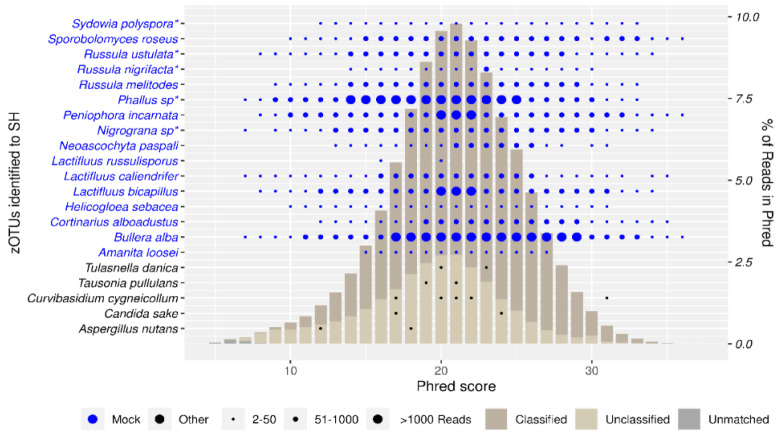
Mock community recovered at different Phred scores using SH approach. * Reference sequences added to database.

Taxon recovery at each Phred score is depicted in Figure 3. Our mock community showed high recovery, with all taxa (excl. *Lactifluus russulisporus* Dierickx & De Crop (2019)) recovered at Phred scores up to Q27. *L. russulisporus* is detected at Q16 (3 reads) and Q20 (2 reads), showing that it was picked up in low abundances. Next to our 16 mock taxa, 28 contaminant SHs were picked up, most of which are singletons.

#### 3.2.3. Bark Substrate Community

The OTU approach produced 2431 OTUs out of 332,874 reads (9.4% of total reads), while the SH approach used 758,755 reads (24.4%) to recover 901 species hypotheses, including two contaminants and 62 singleton SHs.

**Figure 4:**
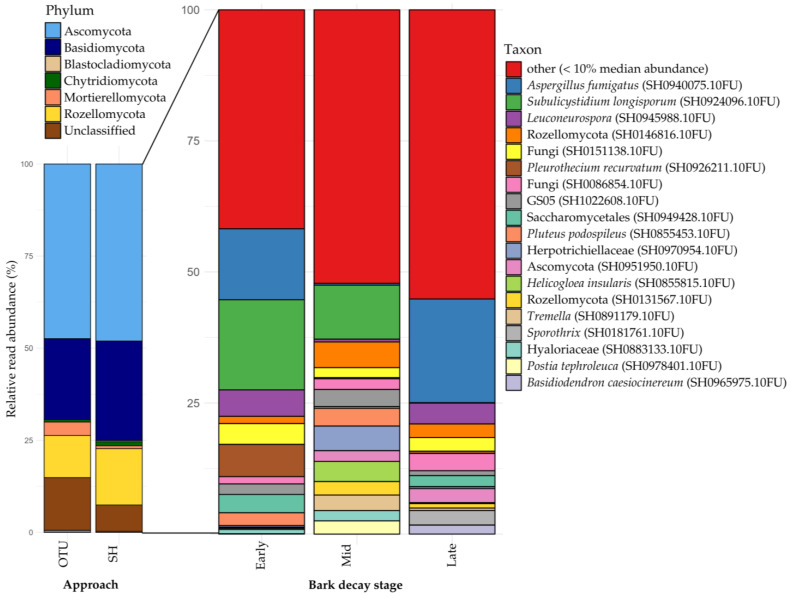
Left: Taxonomic composition across approaches. Right: Composition in each bark decay stage for the SH approach. Phyla with median abundance < 1% not shown (Glomeromycota, Olpidiomycota, Kickxellomycota, Zoopagomycota, Mucoromycota, Entomophthoromycota, Sanchytriomycota, Aphelidiomycota, and ‘GS01_phy_Incertae_sedis’).

At the phylum level, recovery of both methods was similar, with most taxonomic units belonging to Ascomycota, then Basidiomycota and Rozellomycota (Figure 4). The OTU approach produced more units that belonged to Mortierellomycota and more units that could not be classified. The SH approach recovered relatively more Basidiomycota and Rozellomycota. Because the main target of our study was to identify ectomycorrhizal taxa, most of which we assume to be recorded in the UNITE database for our study area, the metabarcoding results and discussion focus mostly on our SH approach.

The inner bark samples were composed of 778 SHs. *Subulicystidium longisporum* (SH0924096.10FU) is the most common SH, with high abundance in both early and intermediate bark decay stages (Figure 4). 19 SHs represent the most common taxa, most of which have no associated species name, for example, two Rozellomycota SHs (SH0131567.10FU and SH0146816.10FU) (Figure 4).

Species richness (SR) increased with bark decay from 106 ± 21 SHs in early decay to 125 ± 27 and 135 ± 28 SHs in intermediate and late decay stages, respectively. Observed and Shannon diversity differed significantly between early and late bark decay stages (Tukey’s HSD, *p* = 0.02 and 0.05, respectively) but not between other pairs. Simpson diversity was similar for all bark decay stages. While richness patterns were consistent in both approaches, significance was more pronounced in the OTU approach.

**Figure 5:**
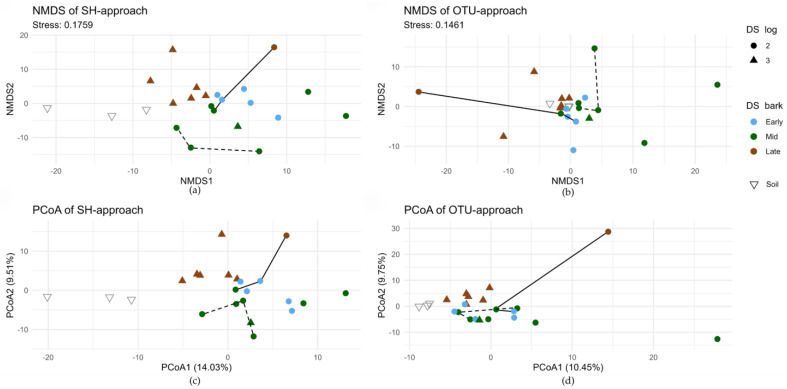
Ordination of both approaches on Aitchison distances. Samples originating from the same logs are connected by the same line types. (**a**) NMDS of SH approach; (**b**) NMDS of OTU approach; (**c**) PCoA of SH approach; (**d**) PCoA of OTU approach.

Both NMDS and PCoA ordination reveal clustering of samples according to the bark decay stage (Figure 5), with the most overlap between early and intermediate bark decay stages.

A notable exception is the NMDS on OTU approach, which shows little data structure with decay (Figure 5b). Two outlier samples (ZF321_1 and ZF327) can be identified in the PCoA of OTU approach (Figure 5d). ZF321_1 is dominated by a single OTU, mapping to a species of Saccharomycetales (66% sample reads). ZF327 contains an OTU that does not occur in any other sample, identified as *Pluteus podospileus* (SH0855453.10FU). This SH is present across multiple samples in the SH approach, thereby not producing an outlier by common absence. As explained before, for two logs, three separate composite samples were taken at several meters distance on the same log. These samples from the same log exhibit greater dissimilarity in ordination space than those from different logs, as illustrated in (Figure 5). Stepwise model selection indicated that the bark decay stage is the best predictor of community composition. PERMANOVA analysis showed that the bark decay stage explains 15.60% of the variance (*p* = 0.001). The effect of the log decay stage was less pronounced, explaining an additional 7.41% (*p* = 0.005). The presence of regeneration (*p* = 0.4) was a non-significant predictor.

In the OTU approach, 50.18% of reads could not be assigned at the species level, including many large clusters. The two largest of these OTUs account for 7% of reads, and both belong to Saccharomycetales. Another 4.5% of reads can be assigned to three OTUs in the Sordariales. Other large OTUs belong to *Trichoderma* (1.6% reads), *Mortierella* (1.4% reads), and Helotiales (1.3% reads).

Fidelity analysis identified 27 and 65 taxa in the SH- and OTU approach, respectively. Results at the 0.01 significance level are presented in Table 2. Of the OTUs identified at the SH level, 18 were shared with those identified by the SH approach. Additionally, 14 SH-level high-fidelity OTUs were exclusive to the OTU approach, with only one (*Rhinocladiella*, SH0970950.10FU) surpassing the 0.01 significance threshold. In addition, indicator analysis (Indval.g function) corroborated these high-fidelity taxa as indicators at the 0.01 level and additionally identified *Pleurothecium recurvatum* (SH0926211.10FU) as an indicator for early bark decay across both approaches.

#### 3.2.4. Mycorrhizae in Decaying Beech Inner Bark

**Figure 6:**
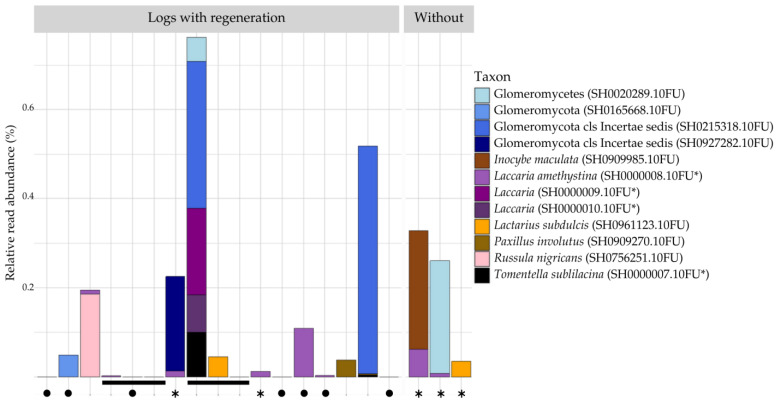
Relative read abundance of EcM species in bark according to presence of regeneration. Bars indicate samples from the same log. Circles indicate logs where EcM were found by morphotyping. Large asterisks under bars indicate samples not used in morphotyping. Sample names from left to right: ZF031, ZF302, ZF311, ZF319_1, ZF319_2, ZF319_3, ZF320, ZF321_1, ZF321_2, ZF321_3, ZF322, ZF323, ZF324, ZF325, ZF326, ZF327, ZF‘681’, ZF317, ZF328, and ZF401. * dummy SH codes.

Our SH-dataset was filtered for reads of mycorrhizal taxa, which were low in abundance. Their relative read abundances in each sample are visualized in Figure 6. Ectomycorrhizal SHs accounted for slightly fewer reads than endomycorrhizal SHs (1.18% and 1.4% respectively). In soil, 3.6% of reads are considered mycorrhizal. Four Glomeromycotan and six ectomycorrhizal taxa are identified: *Lactarius subdulcis* and *Russula nigricans* (Russulaceae), *Inocybe maculata* (Inocybaceae), *Paxillus involutus* (Paxillaceae), *Tomentella sublilacina* (Thelephoraceae), and three *Laccaria* species (Hydnangiaceae). Two of these were detected as large *Laccaria* zOTUs, containing 36 and 12 sequences in our SH approach, but failed to align with our included reference sequence for *Laccaria amethystina*. At the same time, they BLASTn match with more than 99% identity to different *Laccaria* taxa. The pairwise distance between these three sequences ranges from 1.4 to 2.2%. Consequently, we incorporated these zOTUs into our reference database (SH0000009-10.10FU) and conducted a re-analysis with the updated database. These two additional references are only present in a single sample (ZF321_1), while *Laccaria amethystina* occurs in six other samples.

We detected no correlation between the presence of regeneration on logs and the number of mycorrhizal reads (r = −0.16). PERMANOVA on mycorrhizal taxa confirmed that the presence of regeneration (R^2^ = 0.08, *p* = 0.47), the decay stage of bark (R^2^ = 0.11, *p* = 0.75), and the decay stage of the log (R^2^ = 0.03, *p* = 0.93) did not have significant effects on mycorrhizal community composition.

## 4. Discussion

### 4.1. Minimum Quality Evaluation

We evaluated Nanopore sequencing for fungal metabarcoding using the full-length ITS region. We defined acceptable read quality as the point where taxonomic unit recovery stabilized, indicated by consistent recovery rates for 98% OTUs (OTU approach) or zOTUs aggregated into SHs (SH approach). While we acknowledge that annotation accuracy might improve with even fewer sequence errors, we believe such changes would be minimal and unlikely to impact biodiversity patterns in community ecology studies.

Beta-diversity comparisons of OTU tables from Q24 and higher-quality reads showed minimal differences, supporting the robustness of this threshold (Figure S2). Furthermore, the difference in OTU numbers with and without singleton exclusion remained stable within this range, indicating consistent clustering. As illustrated in Figure 2b, the number of recovered 98% OTUs declines rapidly when sequencing accuracy exceeds the clustering threshold (Q17 for 98% OTUs) and stabilizes at Q24–25, where errors minimally impact clustering.

The SH approach bypasses Phred-based filtering by prioritizing read classifiability over average base call quality. Surprisingly, the proportion of classified reads is only partly correlated to the Phred score, with a strong increase at the low end of quality up to Q16, especially in more complex datasets like our bark community (Figure S3). For higher Phred scores, classifiability is independent of the average Phred score. Therefore, beyond Q16, the average Phred score does not reliably predict classifiability.

Our experimental results demonstrate that both the SH- and OTU approach are viable and complementary due to their distinct quality filtering methods. The SH approach avoids signal loss from Phred score filtering and does not blur biological signals by predefining clustering thresholds, using mapping confidence as the quality filter instead. Because SH recovery remains stable regardless of Phred score, also when singleton zOTUs are included, the SH approach eliminates the need for Phred score filtering and singleton zOTU exclusion

Our results show that raw Nanopore data, when appropriately filtered, can support a traditional metabarcoding approach without needing to generate consensus sequences or use complex protocols like Unique Molecular Identifiers [68,69] or Rolling Circle Amplification [70]. We recommend filtering at Q25 for 98% OTUs and to re-evaluate for other clustering thresholds or marker genes. While this currently still loses much data, it effectively identifies larger OTUs, likely key drivers of ecological patterns. Moreover, this threshold can be maintained with expected improvements in Nanopore data quality [71].

While the presented methods of quality filtering are effective, there remains an unoccupied space for more sophisticated techniques. Projecting the zOTU trend (Figure 2a) suggests that Phred scores of >Q30 are necessary for zOTUs to serve as a reliable analysis unit. If such a quality threshold is reached, it opens up the possibility to use Amplicon Sequence Variants (ASVs) with DADA2-like error correction techniques, which assumes that the data contain a non-trivial fraction of error-free reads [72].

### 4.2. Mock Community

Both approaches on the mock community yielded similar results in terms of identified taxa. The OTU approach’ shortcomings of both low data retention and a priori clustering are obvious, with *Lactifluus russulisporus* filtered out due to low abundance (no presence at ≥Q25) and *Russula nigrifacta* and *R. ustulata* being merged to a single taxon (*R. ustulata*) as they differ less than the clustering threshold.

In contrast, the SH approach demonstrated a higher resolution, with 46% of zOTUs being confidently mapped to SH, accounting for 75% of total reads (Figure S3). Classification rates increased rapidly up to a Phred score of Q16, confirming that even modest-quality reads can be reliably classified.

The taxon recovery graph (Figure 3) reveals that nearly all taxa were effectively recovered at Phred scores up to Q27. We attribute the low recovery of *L. russulisporus* to poor DNA extract quality and PCR bias [48]. Despite expecting some false positives, our analysis found no misidentifications, demonstrating the robustness of our classification thresholds.

Our study demonstrated the effectiveness of the SH approach, rather than the OTU approach, in accurately identifying closely related taxa. This is shown by the consistent detection of all species from the included *Lactifluus* and *Russula* groups across various Phred scores. We did not pick up any false positives; for example, no zOTUs matched the reference sequence for the closely related *R. ambusta*.

We recommend the routine use of mock communities as positive controls. Their use offers a low-effort strategy to evaluate both qualitative and quantitative aspects of sequencing runs. A more sophisticated mock should include taxa that are not present in the sample’s locality to ensure that index-switching and cross-contamination can be attributed to the control [1]. An interesting extension of this concept are non-biological, synthetic spike-in controls, like SynMock, which mimic biological diversity without the risk of overlapping with natural taxa [73].

### 4.3. Inner Bark Community

Both approaches classified less than a quarter of the bark dataset reads. While the SH approach still manages to use more than double the amount of reads than the OTU approach, it is obvious that in complex and natural communities, many reads cannot be matched to a database confidently. Even with high-quality reads (Q28), only 36% of reads (31% of zOTUs, Figure S3) were identifiable at the SH level.

At the phylum level, both approaches produced broadly similar outcomes (Figure 4). However, the OTU approach detected a notably higher proportion of Mortierellomycota, largely due to two large OTUs (*Linnemannia amoeboidea,* 4099 reads; *Mortierella* sp., 3058 reads). In contrast, the SH approach yielded a greater recovery of Basidiomycota and Rozellomycota. The subtle nature of these differences suggests both approaches adequately capture diversity. Unless stated otherwise, the species-level results discussed hereafter pertain to the SH approach.

In our bark samples, across decay stages, we found a co-dominating mix of typical soil (e.g., *Aspergillus fumigatus*) and wood-inhabiting fungi (e.g., *Subulicystidium longisporum*, *Pluteus podospileus*, and *Postia tephroleuca*). This mix reflects our characterization of the substrate as ‘soil-like wood’. A significant number of reads were assignable to taxa capable of growing as yeasts (Saccharomycetales) and known mycoparasites (*Tremella*), alongside some less clearly defined taxa such as the enigmatic Rozellomycetous ‘GS05’ and a Hyaloriaceae SH. Interestingly, the latter is also present in the OTU approach, and a manual BLASTn search suggests these sequences likely originate from *Myxarium podlachicum* (Bres.) Raitv. (1971), a common jelly fungus in the reserve. Interestingly, members of our lab have described a mycoparasite inhabiting this species, *Slooffia micra* (Bourdot & Galzin) (Schoutteten 2023) [74], which, despite targeted efforts, has not been found in the sampled area. *S. micra* was incorporated in the latest version of UNITE [53]) as SH0867261.10FU and was recovered in relatively high abundance (213 and 64 reads in the SH- and OTU approaches, respectively). This surprising result hints that this species has part of its life cycle outside of its host basidiomes (fruitbodies were excluded from samples). Indeed, *Slooffia* spp. have been isolated as yeasts from soils, litter, and insect feces [74]. Nevertheless, at the time of writing, it had not been observed in eDNA samples on UNITE. Additionally, several species typically associated with beech deadwood, such as *Simocybe centunculus*, *Coprinellus micaceus,* and eight species of *Pluteus,* were found. Strangely, *Mycena* species, expected to be diverse and abundant, were conspicuously absent from our bark dataset. This is particularly striking given that *Mycena galericulata* and *M. haematopus* are among the most prevalent fungi on deadwood in the reserve [75,76]. The findings outlined in this paragraph highlight the added value of solid field knowledge when interpreting eDNA data.

We observed significant changes in species richness and composition corresponding to bark decay. Species richness (SR) and Shannon diversity metrics varied between early and late decay stages but never with intermediate decay. This pattern suggests that early and late decay stages represent opposite ends of a decay continuum characterized by a gradual species gain and turnover. As the bark decays, it becomes more soil-like, as demonstrated in Figure 5. However, it does not fully converge to resemble the soil community. We attribute this to the presence of colonization barriers and greater environmental fluctuations, such as variations in temperature.

The bark decay stage best explains variation in beta diversity, accounting for 15.6% of the variation, while a model incorporating both the bark and log decay stage explains approximately 23% of the variation. This suggests that the decay gradient is a primary determinant of community composition and aligns with patterns in coarse woody debris, where fungal communities vary significantly between decay stages due to shifting abiotic conditions attracting different specialized species [21,76,77]. We observed high community turnover, with intra-log communities exhibiting greater beta-diversity than those across different logs (Figure 5). This suggests that our sampling was insufficient to capture the bark community at the log level, and we hypothesize that bark volume is an important factor for community richness, provided an adequate surface is sampled.

Both fidelity and indicator analyses underscore the utility of the OTU- and SH approaches in pinpointing critical taxa, with all but one SH-level taxon being shared at a significance level of *p* ≤ 0.01 (Table 2). Simultaneously, these analyses also emphasize the importance of incorporating OTUs at all taxonomic levels, as OTU-only indicators were detected at each stage of bark decay (early: *Tulasnella*; mid: Hydnodontaceae, *Rhinocladiella*, and Helotiales; late: Rozellomycota, *Trichoderma*, and Hypocreales).

The identification of decay stage-specific indicators further underpins the successional nature of bark fungal communities.

### 4.4. Ectomycorrhizal Colonization of Beech Deadwood

In the morphotyping analysis, around half of the investigated saplings growing on logs were colonized by EcM fungi. EcM coverage, species diversity, and the proportion of colonized saplings were significantly lower compared to the saplings growing in soil. Only two species could be identified forming associations with the saplings growing on deadwood: *Laccaria amethystina* and *Tomentella sublilacina*. *Tomentella* is a multistage, highly competitive ECM genus in mature forests [78]. *T. sublilacina* is a corticoid species forming basidiomes on wood and was also identified by Tedersoo et al. [25,79] on saplings growing on decaying logs of *Betula pendula* Roth (1788), *Picea abies* and *Nothofagus cunninghamii* (Hook.f.) Oerst (1871). Baldrian et al. [17] metabarcoded course woody debris in a beech-dominated forest and found the species to be the second most common EcM at 0.45% of reads. Interestingly, only one sapling was colonized with both *L. amethystina* and *T. sublilacina*, which hints at competitive exclusion. Indeed, *T. sublilacina* has been found to be capable of competitive exclusion of, e.g., *Tylospora fibrillosa* (Burt) Donk (1960) [25] and *Lactarius* spp. [26].

*Laccaria amethystina* is considered a late successional species In Europe [80] and can form basidiomes on decaying wood of beech [76], Norway spruce [81] and Silver fir [82]. This species produces small, short-lived genets dedicated to the production of meiospores [83]. Moreover, it prefers to associate with beech saplings within our study area [84]. Supporting this, Grebenc et al. [85] found a positive association between the occurrence of *L. amethystina* and the number of beech seedlings, and Hortal et al. [86] demonstrated that beech roots can be simultaneously colonized by multiple *L. amethystina* genets. These characteristics underscore its classification as an R-strategist, adept at spore production and dispersal, which explains its propensity to rapidly colonize new microsites within its late successional habitat.

Despite the relatively low abundance of mycorrhizal reads, several mycorrhizal SHs were detected. Ectomycorrhizal (EcM) species accounted for 1.18% of the total reads, while endomycorrhizal species made up a slightly higher proportion at 1.4%, as illustrated in Figure 6. We visually confirmed the presence of arbuscular mycorrhizal fungi by staining *Acer* saplings growing on certain beech logs (Personal oberservation, staining method according to Vierheilig et al. [87]). Supporting our observations, Baldrian et al. [17] found EcM fungi in similarly low abundances across early and intermediate log decay stages of beech, only becoming more abundant in late log DS. Our analysis revealed no significant correlation between the presence of logs with and without regeneration. This suggests that while specific mycorrhizal taxa are effectively colonizing decaying bark, their distribution is independent of sapling presence. The absence of correlation indicates that other environmental or biological factors have a more pronounced impact on shaping these mycorrhizal communities. Our metabarcoding results offered limited insights into the presence of ectomycorrhizal (EcM) fungi in deadwood. In contrast, morphotyping is per definition more detailed as it also demonstrates successful establishment. Like the results of our morphotyping analysis, EcM read abundance and diversity are higher in soil than in bark, with EcM diversity in bark being a subset of that in soil. Thus, both methods indicate that soil is the more beneficial habitat for mycorrhization.

Across our analyses, *L. amethystina* and *T. sublilacina* are identified as the key EcM in bark. In terms of EcM establishment, two critical factors can be identified: the source of the inoculum (via propagules or mycelium emerging from the soil) and the timing of arrival (before or after seedlings arrive). Mycelia in the soil may respond to the presence of deadwood by initiating growth into it. Initially, wood is resistant to penetration, but inner bark, with its softer consistency and quicker degradation, may serve as an early colonization pathway for soil-based EcM. Another possibility is that mycelium grows out of the soil regardless, as some fungi, such as *Tomentella* spp., are known to use wood as a substrate for fruiting. Alternatively, EcM can establish within deadwood bark through propagules, deposited either from the air or vectored by arthropods. The latter route benefits from direct deposition into the decaying substrate, a process well-supported by studies demonstrating the effectiveness of arthropods as fungal vectors [88,89,90].

While EcM are able to colonize deadwood, their viability in the absence of seedlings remains uncertain. Without seedlings, deadwood could act as a refuge from intense competition in the soil among well-established mycelia linked within the EcM network, especially among mature beeches. Such EcM may utilize delignified carbohydrates as a carbon source while awaiting their symbiotic partners [91,92], although such capabilities are now questioned [23]. Still, several studies suggest that certain EcM can contribute to decomposition [93,94]. This process is likely not targeted at carbon acquisition but rather helps mobilize nitrogen from organic matter [23,95], which could enhance seedling establishment. Thus, the formation of common mycelial networks prior to the arrival of saplings could explain the enhanced sapling establishment sometimes observed in these specific forest microhabitats [25]. Alternatively, successful EcM establishment might require germination in the presence of roots, raising questions about whether the EcM reads detected in logs without seedlings are from spores, mycelia doomed to perish, or necromass. Our data present mixed signals. First, there is no evidence that EcM accumulation correlates with the bark decay stage (DS), suggesting either that establishment is difficult after early bark DS or, more likely, that EcM do not accumulate in time. Second, the absence of a signal of beech regeneration on mycorrhizal reads indicates that they occur independently of their partners. This dual pattern supports our belief that colonization can happen in the absence of seedlings, but forming symbiosis is crucial for the continued survival of mycorrhizae in decaying bark. Mechanistic experiments (e.g., Fukasawa and Kitabatake [96]) or sampling forests without natural regeneration would enhance our understanding.

### 4.5. Database

By prioritizing the classifiability of our data within the SH framework, our study demonstrates that sequences, even those with moderate average quality, can be accurately classified. User-defined references can also be included if existing SHs are inadequate, as demonstrated in our analysis of mock and bark samples. For example, we added references for mock and ectomycorrhizal taxa that could not be confidently assigned to a SH. Unfortunately, incorporating such references also hampers cross-study communication of taxa. In that regard, the nascent SH-matching tool [97] deserves further attention as it makes the public assignment of new SH possible, but we did not extensively test it.

We assumed EcM in our sampling area to be well documented in the database, and indeed, many reference sequences are available for, e.g., *Laccaria amethystina*. Nevertheless, we encountered difficulties in mapping zOTUs of *Laccaria* and *Tomentella* to SH, as well as issues with the interpretation of some taxon names in SHs with many associated voucher sequences. These problems may stem from the proliferation of sequence data and inconsistent sequence annotations, which complicate the precise definition and annotation of SHs. Addressing these challenges will require manual curation, a daunting task at the scale of UNITE.

We recommend leveraging reference-based approaches, such as the SH approach, in well-characterized communities, i.e., in regions or substrates that have been extensively sampled. A significant benefit of this approach is superior data retention and aggregation at the SH level, facilitating communication and comparisons of taxonomic units across analyses and studies and enhancing reproducibility.

### 4.6. Nanopore Metabarcoding

We found Nanopore to be a suitable platform for metabarcoding, although currently, only a fraction of the generated data meet the quality required for broad-scale adoption. Despite these limitations, the platform offers unique advantages beyond the elimination of traditional read length constraints, making it particularly advantageous in the field of mycology, which heavily involves citizen scientists [98,99]. The low initial capital investment and the presence of an active user community contribute to its accessibility, which makes it a practical option for both amateurs and smaller research groups. Moreover, the ability to conduct in-house sequencing eliminates the need for outsourcing, thereby providing autonomy over workflows and data, empowering especially citizen scientists.

The Nanopore metabarcoding field is experiencing rapid growth, with a variety of tools and analysis strategies being developed. We built eNano to be a simple-to-understand tool with straightforward integration of standard metabarcoding steps. Therefore, it is not designed to outperform emerging tools but rather to serve as a valuable baseline for comparison. The simple installation process (a single binary file) and the clarity of the procedures make eNano also suitable for educational purposes.

Considering the dynamic nature of this field, we recommend that researchers also explore other strategies like implementing Unique Molecular Identifiers [68,69] or tools such as EMU [100] and PIMENTA [101]. Noteworthy alternatives such as Decona [13] and CONCOMPRA [15] do not require reference databases to generate taxonomic units and have been demonstrated to be viable alternatives [11,15,102]. Regardless of the approach, benchmarking tools should ideally include a baseline for comparison in the form of a properly quality-filtered OTU table generated using the OTU approach.

## 5. Conclusions

This study evaluates the effectiveness of Nanopore sequencing for fungal metabarcoding using the full-length ITS region through the analysis ofboth a mock and natural community. We demonstrate that both the reference-based SH approach (mapping reads to a database) and the OTU approach (de novo clustering) are viable options. The SH approach ensures stable taxonomic unit recovery and is only minimally impacted by low Phred scores (<Q16) when strict mapping criteria are applied. The OTU approach is feasible but requires stricter quality filtering, which currently results in significant data loss. However, it is capable of identifying taxonomic units that are not represented in existing databases. We recommend using reads with a quality score of ≥Q25 when constructing 98% OTUs.

Our analysis of fungal communities in decaying bark emphasizes the decay gradient as a primary determinant of community composition. Ectomycorrhizal fungi are found to be less diverse and abundant in bark compared to soil, indicating that soil provides a more favorable environment for mycorrhization. Despite this, *Laccaria amethystina* and *Tomentella sublilacina* are identified as key species colonizing decaying bark and capable of successful establishment. These findings suggest that decaying bark can serve as a microhabitat for specific mycorrhizal species, providing a potential explanation for the presence of mycorrhizae, which are observed in early decay stages in other metabarcoding studies on deadwood.

We introduce the eNano tool, which offers a simple baseline approach to Nanopore metabarcoding and is suitable for broad adoption. Overall, Nanopore offers a promising platform for fungal metabarcoding, with the potential to yield valuable ecological insights and engage a wider audience of researchers and citizen scientists. As read quality continues to improve, the use of Nanopore data in a more classical metabarcoding fashion, whether through reference databases or by constructing OTUs, is likely to take hold.

## Figures and Tables

**Table 1:** Decay stages of inner bark layer in European beech (Fagus sylvatica) deadwood. Note that bark decay stage (DS) may differ within the same log.

Bark Decay Stage	Description
Early	Layer (incl. inner bark) is difficult to penetrate with a metal spoon (force needed), and almost all fibers are intact and dark yellow in color. This corresponds to log decay stage (DS) 1 or early 2.
Intermediate	Layer crumbles and can be scooped up with a metal spoon. They crumble with minimal force between fingers, some fibers intact, light brown in color. This corresponds to a late log DS 2 or early 3.
Late	Layer is almost fully decayed, penetrable without force; it easily disintegrates between fingertips–resembles soil, no fibers intact, (dark) brown in color. Corresponds to a late log DS 3 or 4.

**Table 2:** High-fidelity taxa for the OTU- and SH approaches that met a significance threshold of *p* < 0.01. Fidelity scores are those from SH approach if taxon is present in both approaches.

DS Bark	Lowest Taxon Name	Fidelity	*p*	SH|OTU	
Early	Chaetosphaeriaceae (SH0980871.10FU)	0.798	0.001	●	●
	*Tulasnella* (OTU 1097)	0.728	0.003		●
	Sordariales (SH0840221.10FU)	0.779	0.008	●	●
	*Pleurothecium recurvatum* (SH0926211.10FU) *	0.950	0.007	●	●
Mid	*Ganoderma adspersum* (SH0762773.10FU)	0.839	0.001	●	
	Hydnodontaceae (OTU 448)	0.729	0.004		●
	Herpotrichiellaceae (SH0970954.10FU)	0.751	0.005	●	●
	*Rhinocladiella* (OTU 75|SH0970950.10FU)	0.697	0.005		●
	Helotiales (OTU 474)	0.729	0.006		●
	Mortierella (SH0960682.10FU)	0.745	0.008	●	●
	*Serendipita* (SH0743656.10FU)	0.730	0.008	●	●
	*Chaetosphaeria decastyla* (SH0980872.10FU)	0.655	0.010	●	
Late	Hyaloscyphaceae (SH0973189.10FU)	0.707	0.006	●	●
	Rozellomycota (SH0910702.10FU)	0.707	0.006	●	
	*Ilyonectria mors-panacis* (SH1450888.10FU)	0.707	0.007	●	
	Rozellomycota (OTU 1883)	0.711	0.007		●
	Pezizomycotina (SH0755218.10FU)	0.808	0.008	●	●
	*Trichoderma* (OTU 4045)	0.707	0.008		●
	Hypocreales (OTU 2917)	0.707	0.009		●
	Leotiomycetes (SH0948543.10FU)	0.707	0.010	●	●

* Additional taxon identified using Indval.g function in multiipatt (indicspecies 1.7.13); fidelity value is the indicator statistic.

## Data Availability

The original contributions presented in the study are included in the article and Supplementary Materials. Sequence data can be accessed as fastq files at the NCBI Sequence Read Archive under BioProject ID PRJNA1151640.

## Supplementary Materials

The following supporting information can be downloaded at https://www.mdpi.com/article/10.3390/jof10100708/s1, Figure S1. Density histogram of SINTAX confidence values at SH-level; Figure S2. Heatmaps of Beta diversity in Bray–Curtis distance between subsampled Phred-specific datasets; Figure S3. Classification rates of zOTUs in SH-approach; Table S1. Overview of sampled logs; Table S2. Mock community composition; Table S3. Mycorrhization and morphotyping results; Table S4. Sanger sequencing results of root tip morphotypes; File S1. OpenDocument Spreadsheet with OTU-tables.
