# Supplementary material for: What Quality Suffices for Nanopore Metabarcoding? Reconsidering Methodology and Ectomycorrhizae in Decaying Fagus sylvatica Bark as Case Study
Source: J Fungi (Basel). 2024 Oct 10;10(10):708. doi: 10.3390/jof10100708 (PMC11508852; doi:10.3390/jof10100708)
Supplement: Supplementary file 1 [file jof-10-00708-s001.zip › supplementary_reviewed.pdf]

Table S1. Overview of logs selected for sampling. Log id identifies deadwood object, metabarcoding and morphotyping indicate analysis type, seedlings denotes presence or absence of seedlings, DS log according to Renvall 1995; DS bark according to Table 1.

Log ID	meta-barcoding	morpho-typing	re-generation	DS log	DS bark
ZF031	+	+	+	3	Late
ZF302	+	+	+	3	Late
ZF311	+	+	+	3	Late
ZF319_1	+		+	2	Mid
2	+	+	+	2	Mid
3	+		+	2	Mid
ZF320	+	-	+	3	Mid
ZF321_1	+		+		Late
2	+	+	+	2	Early
3	+		+		Mid
ZF322	+	-	+	3	Late
ZF323	+	+	+	3	Late
ZF324	+	+	+	2	Early
ZF325	+	+	+	2	Mid
ZF326	+	+	+	2	Mid
ZF327	+	+	+	2	Mid
ZF'681'	+	+	+	3	Late
ZF328	+	-	-	2	Early
ZF401	+	-	-	2	Early
ZF317	+	-	-	2	Early
ZF037	-	+	+	4	Late
ZF060	-	+	+	4	Late
ZF076	-	+	+	4	Late
ZF109	-	+	+	3	Late
ZF112	-	+	+	4	Late
ZF300	-	+	+	3	Late
ZF306	-	+	+	3	Late
ZF307	-	++	+	3	Late
ZF310	-	+	+	3	Late
ZF'1026'	-	+	+	3	Late
OKVZF02	-	+	+	4	Late

Table S2. Mock community composition.

Taxon	Herbarium nr.	Accession nr.	Motivation for inclusion
Russula nigrifacta	RDL 16-044	MW172308 [2]	EcM, sensitivity at species complex (to *R. ustulata*)
Russula ustulata [1]	AV 16-019	MW172312 [2]	EcM, sensitivity at species complex (to *R. nigrifacta*)
Russula melitodes	RDL-37-03-09-2014	PP768987	EcM, sensitivity at subgenus (to *R. nigrifacta*, *R. ustulata*)
Lactifluus russulisporus	REH 9389	KR364097 [2]	EcM, sensitivity at subgenus (to *L. caliendrifer*)
Lactifluus caliendrifer	KW 387	MK517655 [2]	EcM, sensitivity at subgenus (to *L. russulisporus*)
Lactifluus bicapillus	EDC 14-249	MH549203 [2]	EcM, sensitivity at genus
Amanita loosei	EDC 14-088	PP768996	EcM, phylogenetic diversity (Agaricales)
Cortinarius alboadustus	AdH 11058	PP768988	EcM, phylogenetic diversity (Agaricales)
Peniophora incarnata	MJD 20-030-C3	PP768995	Phylogenetic diversity (Russulales)
Helicogloea sp.	NS 18-1302	MK908009 [2]	Phylogenetic diversity (Atractiellales)
Neoascochyta paspali	ID 7126-C1	PP768994	Phylogenetic diversity (Pleosporales)
Nigrograna sp. [1]	ENZ 20-071-C6	PP768990	Phylogenetic diversity (Pleosporales)
Phallus sp. [1]	JL 18-001	PP768991	Phylogenetic diversity (Phallales)
Sydowia polyspora [1]	RGMYCO Chalet-7	PP768989	Phylogenetic diversity (Dothideales)
Sporobolomyces roseus	NS 19-388-C1a	PP768993	Phylogenetic diversity (Sporidiobolales)
Bullera alba	NS 20-021-C2a	PP768992	Phylogenetic diversity (Tremellales)

[1] *sequences added to reference database;* [2] *sequences not published in this study*

Table S3. Mycorrhization and morphotyping results for the log and soil extracted saplings sampled for EcM screening. Sapling age estimated by counting number of bud scar zones. EcM species: different EcM species identified on the sapling roots. EcM coverage: summed proportion of root tips colonized by these species

Substrate	DS bark	Sapling Age (yr)	EcM species	EcM coverage (%)
deadwood	Mid	2	none (n = 3)	/
		2	*Laccaria amethystina*	0,23
		2	*Laccaria amethystina*	0,2
		2	*Laccaria amethystina*	0,18
	Late	2	none (n = 5)	/
		2	*Tomentella sublilacina*	0,22
		2	*Laccaria amethystina*	0,17
		3	none (n = 1)	/
		3	*Laccaria amethystina*	0,22
		3	*Laccaria amethystina*	0,08
		4	*Laccaria amethystina*	0,28
		4	*Laccaria amethystina*	0,14
		6	*Laccaria amethystina*	0,02
		6	*Laccaria amethystina*	0,24
		6	*Laccaria amethystina, Tomentella sublilacina*	0,3
		8	none (n = 1)	/
		9	*Laccaria amethystina*	0,27
Soil		2	*Cenococcum geophilum*	0,11
		2	*Lactarius subdulcis, Xerocomellus pruinatus, Cenococcum geophilum*	0,39
		2	*Xerocomellus pruinatus, Cenococcum geophilum, Russula ochroleuca, Elaphomyces* cf. *muricatus*	0,15
		4	*Inocybe napipes, Cenococcum geophilum, Scleroderma citrinum, Lactarius subdulcis*	0,44
		4	*Scleroderma citrinum, Elaphomyces* cf. *muricatus, Cenococcum geophilum, Laccaria amethystina, Melanogaster* cf. *intermedius, Russula ochroleuca*	0,6

Table S4. Sanger sequencing results of root tip morphotypes

Blast ID	Herbarium nr.	Accession nr.
Laccaria amethystina	LT22-006	PP804254
Laccaria amethystina	LT22-007	PP804243
Laccaria amethystina	LT22-008	PP804244
Laccaria amethystina	LT22-009	PP804245
Tomentella sublilacina	LT22-011	PP804246
Lactarius subdulcis	LT22-012	PP809298
Xerocomellus pruinatus	LT22-013	PP804247
Xerocomellus pruinatus	LT22-015	PP809299
Russula ochroleuca	LT22-016	PP804248
Tomentella cf. *sublilacina*	LT22-019	PP804249
Inocybe napipes	LT22-022	PP809300
Russula ochroleuca	LT22-024	PP804250
Scleroderma citrinum	LT22-027	PP809301
Melanogaster cf. *intermedius*	LT22-031	PP804251
Melanogaster cf. *intermedius*	LT22-032	PP809302
Russula ochroleuca	LT22-035	PP804252
Russula ochroleuca	LT22-036	PP804253

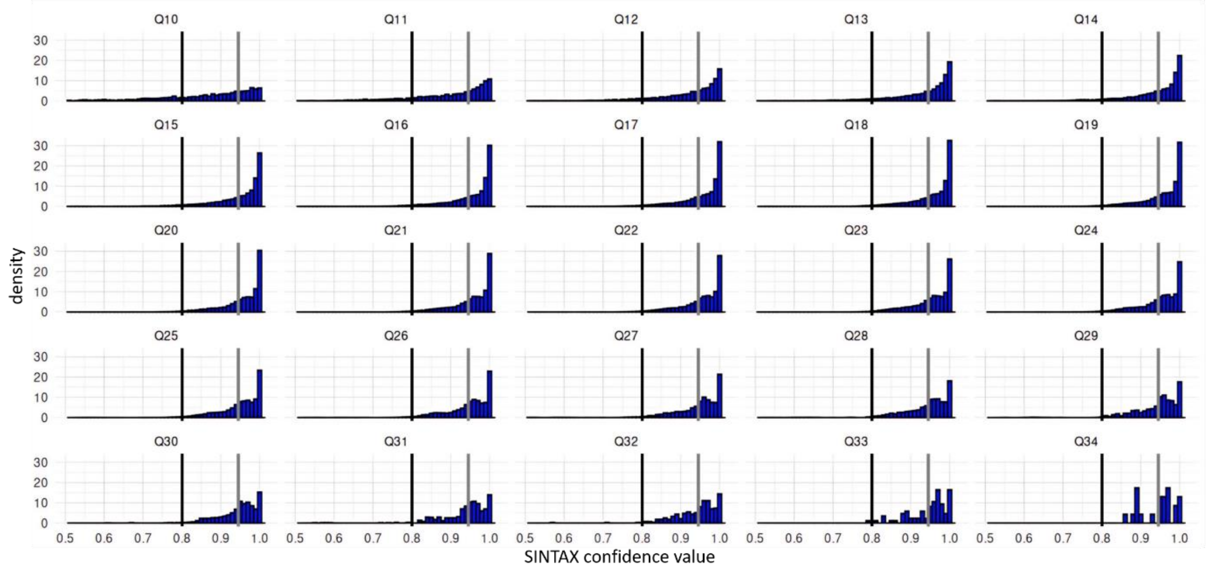

Figure S1. Density histogram of SINTAX confidence values at SH-level in the mock community, arranged per Phred score. Black (0.80) and gray (0.95) bars represent used thresholds multitons and singletons respectively.

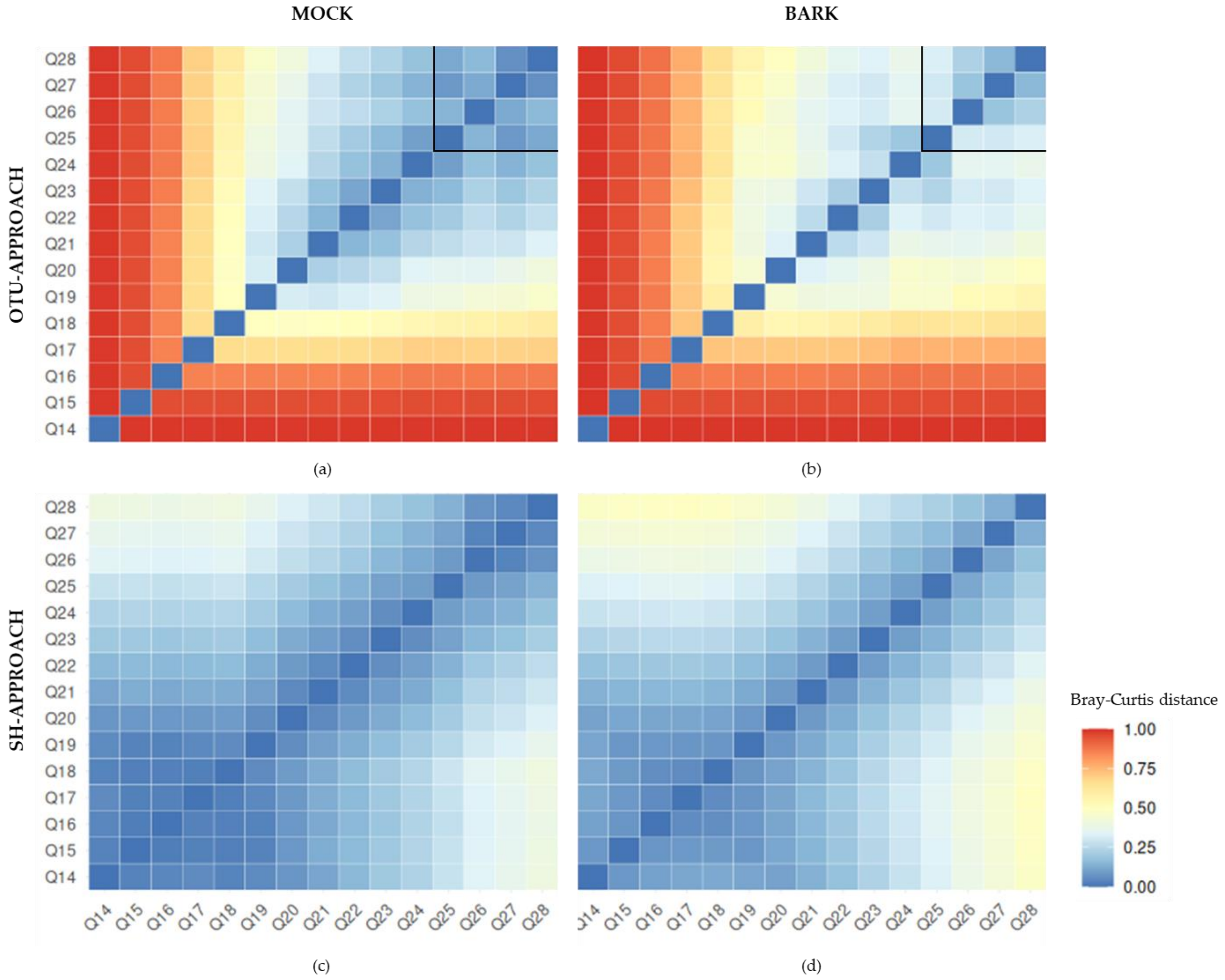

Figure S2. Heatmaps of Beta diversity in Bray-Curtis distance between subsampled Phred-specific datasets. (a) Mock community in OTU-approach (4245 reads/Phred), (b) Bark community in OTU-approach (42560 reads/Phred), (c) Mock community in SH-approach (4245 reads/Phred), (d) Bark community in SH-approach (42560 reads/Phred). Singletons included, black lines indicate quality threshold in the OTU-approach.

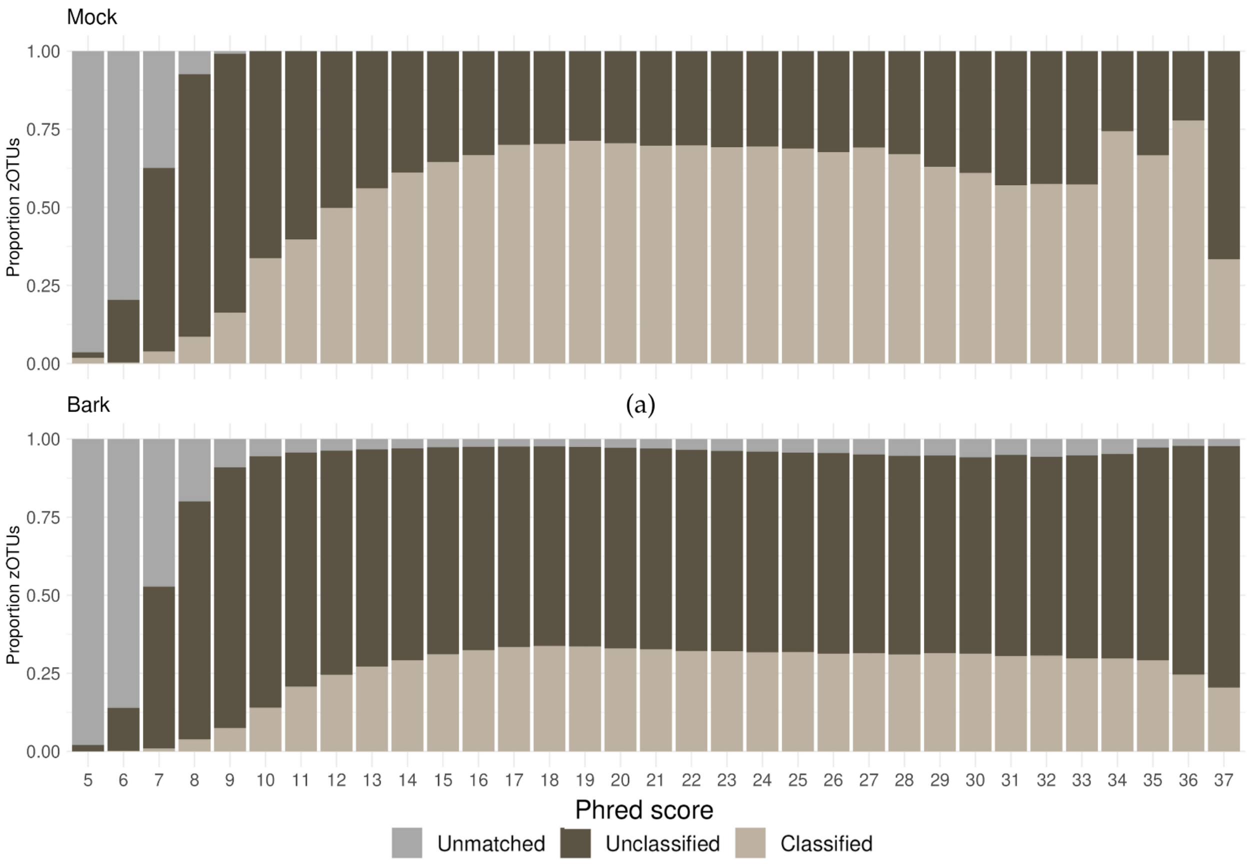

(b)

Figure S3. Classification rates of zOTUs in SH-approach for (a) Mock dataset and (b) Bark dataset. All reads are used and indicated according to Unmatched a the fungal domain level, Unclassified at the SH-level and Classified at the SH-level.
